# Hydro-Therapy

**Published:** 1910-07

**Authors:** L. Amster

**Affiliations:** Atlanta, Ga.


### Journal-Record of Medicine

Successor to Atlanta Medical and Surgical Journal, Established 1855, and Southern Medical Record, Established 1870.

OWNED BY THE ATLATNA MEDICAL JOURNAL CO.

Published Monthly

*Official Organ Fulton County Medical Society, State Examining Board, Presbyterian Hospital, Atlanta, Birmingham and Atlantic Railroad Surgeons' Association, Chattahoochee Valley Medical and Surgical Association, Etc.*

EDGAR G. BALLENGER., M. D., Editor.

BERNARD WOLFF, M. D., Supervising Editor.

A. W. STIRLING, M. D., C. M., D. P. H., J. S. HURT, B. Ph., M. D.

GEO. M. NILES, M. D., W. J. LOVE, M. D., (Ala.); Associate Editors.

E. W. ALLEN, Business Manager.

COLLABORATORS

Dr. W. F. WESTMORLAND, General Surgery.
F. W. McRAE, M. D., Abdominal Surgery.
H. F. HARRIS, M. D., Pathology and Bacteriology.
E. B. BLOCK, M. D., Diseases of the Nervous System.
MICHAEL HOKE, M. D., Orthopedic Surgery.
CYRUS W. STRICKLER, M. D., Legal Medicine and Medical Legislation.
E. C. DAVIS, A. B., M. D., Obstetrics.
E. G. JONES, A. B., M. D., Gynecology.
R. T. DORSEY, Jr., B. S. M. D., Medicine.
L. M. GAINES, A. B., M. D., Internal Medicine.
J. N. LeCONTE, M. D., Disease of the Stomach and Intestines.
L. B. CLARKE, M. D., Pediatrics.
EDGAR PAULIN, M. D., Opsonic Medicine.
THEODORE TOEPEL, M. D., Mechano Therapy.
R. R. DALY, M. D., Medical Society.
A. W. STIRLING, M. D., etc., Diseases of the Eye, Ear, Nose and Throat.
BERNARD WOLFF, M. D., Diseases of the Skin.
E. G. BALLENGER, M. D., Diseases of the Genito-Urinary Organs.

VOL. LVI. JULY, 1910 No. 4.

### HYDRO-THERAPY.

BY L. AMSTER, M. D., ATLANTA, GA.

Although I have been a member of this society for the past 18 years, I am not aware that the subject of hydro-therapy has ever been discussed at its meetings, with the possible exception of the Brand method of treating typhoid fever.

The present status of hydro-therapy makes it a necessity, that every physician be at least familiar with the rudimentary elements of this important therapeutic agent.

For many years the medical profession has been indifferent to the use of water in treating disease, for various reasons:

Hydro-therapy has not been taught in medical eschools. Text-books on medicine, while giving explicit instructions for the administration of drugs, merely mention hydro-therapy being indicated in some diseases.

Hydro-therapy having been in the hands of empirics and charlatans, the medical profession naturally looked upon it with suspicion and distrust, thereby leaving a rich and worthy field in medicine to the exploitation of quacks. Furthermore, the use of hydro-therapy is associated in many physicians' minds with elaborate apparatus and inaccessible for general use. The fact is, that many valuable hydriatic procedures are admirably adapted for home use and just as effective as treatment in especially appointed institutions.

It is no doubt true, that the psychic effect upon patients, seeing elaborate apparatus, handled by skillful attendants, is wholesome and confidence-inspiring; still the success of hydro-therapy lies more in the skillful application, than in the various appliances. Professor Winternitz believes that a sheet and a bucket of water in the hands of a good hydro-therapist are quite sufficient for obtaining most physiological effects of water treatment.

I suppose most of us have an idea that hydro-therapy is a good thing; we order its use in a vague and indefinite sort of a way. We tell our patients to take a cold bath or a warm bath of a douche for the tonic or carminative effect, but we do not know the whys and wherefores of its physiology. The essential part of the treatment we lose sight of. We do not give instructions as to exact temperature, duration, force and other indispensable points. These points are really the factors which bring about the physiological effect; the water per se is simply the medium.

We do not tell our patients to take strychnine, morphine, mercury or even harmless drugs, but prescribe the proper dose, time and mode of administration. This is the only way to get the desired therapeutic effect. It is the same way with water treatment. It is necessary to familiarize ourselves with the principles of hydro-therapy and prescribe intelligently, like we do drugs.

Water is not a panacea; it is productive of much good, and often of much evil. To direct a patient, who is probably a frail, anemic woman, to take cold baths, without proper directions, is wrong. To advise a patient, who may have arterio-sclerosis, or some cardiac or pulmonary trouble, to go and take a Turkish or Russian bath, with no physician at hand, may have very serious consequences. But once we understand the basic principles of hydro-therapy we can expect just as certain and definite results from water treatment as we can from a dose of medicine. The emporical use of water is disappointing and brings hydro-therapy into disrepute.

The history of hydro-therapy is as old as medicine itself. The ancient Egyptians, Greeks and Romans all have used water as a therapeutic agent. The many Roman ruins bear testimony to this day, how much care and attention these people must have devoted to the comfort and luxury of their private and public bath establishments. Since the time of Hypocrates and Galen medical therapeutics has undergone many changes, almost all of it has been abandoned, only diet and the use of water has withstood the test of time.

After the fall of the Roman Empire hydro-therapy was not much used.

In the middle ages France, Italy and England maintained water as a cure. In Germany, due to "Therapeutic Nihilson," a sect of "Nature Doctors" of the type of the priest Kneipp espoused the cause of hydro-therapy. Their irrational and phantastic propaganda alienated the interest of the medical profession towards hydro-therapy. It remained for Priesnitz, an uneducated Austrian peasant during the first quarter of the nineteenth century, to force the world—including the medical profession, to acknowledge the wonderful merits of water treatment.

Though the methods of Priesnitz were crude, such was his ingenuity and his inventive intuition in the application of water baths externally and internally, that many were cured by his treatment. At first denounced by the medical profession, his success was so great as to compel the attention of the best and most scientific physicians of his time. The more liberal minded medical men investigating his claims and being convinced of the great value of hydro-therapy, laid down their aversion and prejudice and accepted it into the fold of medical therapeutics.

Fleury, in France, founded a school of hydro-therapy by the introduction of "douches" as the chief method. France since has steadily advanced in the development and practical application of hydro-therapy. The French government having given it official recognition. The well-known Charcot was an enthusiastic advocate of it.

In England hydro-therapy has not obtained a very firm foothold, in spite of the labors of Currie, who has written one of the best works on the subject.

In America, Dr. Benjamin Rush and Dr. John Bell, both of Philadelphia, were its early advocates. Of late years, Dr. Simon Barnch, of New York, and Dr. J. H. Kellogg, of Battle Creek, are its most noted exponents.

The foremost American physicians have placed the stamp of approval upon hydro-therapy. The New York College of Physicians and Surgeons has made its study obligatory and other schools are falling in line.

To Professor Winternitz, of Vienna, however, medical science owes nearly all it has learned about the scientific uses of water.

After many years of arduous labor and untiring investigation, fortified by physiological, pathological and therapeutic learning, he clearly set forth the scientific truth, that water has certain distinct physiological effects on circulation, on the nervous system, on the skin, on respiration, on metabolism, etc.

By his painstaking investigations he rescued hydro-therapy from Empirics and placing it upon so sound a scientific basis as to command its recognition by the most eminent men for all time to come.

Up to the time of Winternitz, the term hydro-therapy was synonymous with "cold water treatment." Some few believe this to be the case to-day. This is a fallacy. Hydro-therapy includes the application of water, cold or hot, in any form, from solid and fluid to vapor; from ice to steam, internally and externally.

Hydro-therapy does not include, however, the use of mineral waters; this is called balneology.

It is important to bear in mind that hydro-therapy depends upon the *thermic* and *mechanical* action of water in disease. It depends upon the degree of cold or heat and upon the force of the water coming in contact with the body, producing mechanical irritation. It is, therefore, strictly speaking, hydro-thermo-mechano-therapy.

This being the case, the question naturally arises in our minds, why use water? There are other agents productive of thermic and mechanical irritation. There are several reasons why water is the best medium for conveying these effects:

1. Its capability for absorbing and conducting cold and heat.

2. Its flexibility and easy convertability from liquid to solid and gaseous form.

3. The rapidity with which it envelopes small and large portions of the body.

4. Its fluidity, whereby size, form and character of the stream can be directed to any and all portions of the body, making it possible to induce local and general effects with precision.

5. It is the greatest solvent. It holds the blood corpuscles in suspension, promotes chemical changes necessary for absorption and assimilation and assists in the elimination of waste material.

When we consider that water is an essential constituent of the human body, forming about 70% of the entire weight, it is but natural that so important a substance should play an important role in therapeutics also.

In order to obtain a clear and intelligent idea of the real therapeutic results of the thermic and mechanical irritation of water applied to the body, it is necessary to be familiar with the present state of knowledge of the action of hydro-therapy upon circulation, nervous system, respiration, temperature, metabolism secretion and excretion. Only then can we employ hydro-therapy intelligently.

Lack of time prevents me from going into detail regarding this important phase of hydro-therapy. I will, however, devote the rest of this paper to the discussion of the one basic principle upon which the success of hydro-therapy is founded, i. e., reaction—the goal of water treatment.

The first stipulation for the success of any hydriatic procedure is, to be able to bring about satisfactory reaction. The warm glow—e. g.—the feeling of Euphoria or wellbeing and sense of refreshment following the temporary shock of a cold plunge or douche is named "reaction."

Reaction depends upon many conditions and circumstances. It depends upon:

The intensity of the thermic and mechanical irritation, the duration of these irritations, the locality of application, and the individual idiocyncrasy or sensitiveness.

This sensitiveness varies in the healthy and the sick, in the strong and the weak, in the anemic and the plethoric.

Reactive sensitiveness, furthermore, varies at different times of the day; upon the empty or full stomach.

These facts have to be considered, when ordering hydriatic treatment.

Sometimes it is not easy to judge in advance, how an individual is going to react. At such times we order certain mild experimental procedures before letting the patient enter upon the appropriate treatment.

Mapping out a plan of treatment for a patient his circulation and innervation has to be considered.

For nervous, flabby, anemic people cold, invigorating irritants will be ordered, but of short duration. Others may require longer impressions with high tempered water. In others again we may have to exclude certain parts of the body from treatment, while other parts may have to be very actively treated in order to produce the desired reaction.

Temperatures of 92°-97° F. cause no appreciable reaction. This is known as the "Neutral Zone."

To promote reaction we order patients to rest under blankets, if they are weak or suffer from some inflammatory condition. In more vigorous people exercise in the open after hydriatic treatment hastens and intensifies reaction.

The more pronounced the irritation upon the nerves, the more thorough is the production of heat and the more pronounced the reaction.

The warmer the body before taking a cold plunge or douche, the more thorough the reaction and consequent warmth.

It is a rule: The body shall be warm before entering upon a cold procedure.

The effect of thermic irritation is enhanced by mechanical assistance.

Reaction is less and tardy after gradual and prolonged abstraction of heat.

Extremely energetic cooling leads to incomplete and delayed or too intense reaction. Excessive cooling of the body may lead to a state of collapse and must be guarded against.

Short, cold procedures produce the following reaction:

Dilatation of the superficial blood vessels with contraction of the internal vessels.

Redness of skin.

Sensation of warmth.

Sensation of comfort and wellbeing.

Slowing of the pulse with increased tension.

Slower and deeper respiration.

Heating of the skin.

Fall of internal temperature.

Increase of perspiration.

The reactions of hot procedures are:

Constriction of blood vessels.

Pallor of the skin.

Frequent, low tension pulse.

Frequent, superficial respiration.

Lessened perspiration.

Depression of internal temperature.

Diminished nervous and mental irritability.

Drowsiness, muscular weakness and indisposition to muscular effort.

Bearing these laws of reaction in mind, it ought not to be difficult to choose hydriatic procedures and meet therapeutic indications.

The uses of water internally, by enteroclysis and hypodermoclysis I shall discuss at some future time.

In conclusion, I wish to say that it has been my privilege to visit and receive instruction in the Winternitz school of hydrotherapy at the Vienna Polyclinic. It has been my privilege to meet the old veteran, Prof. Wm. Winternitz, who though over 80 years old, still labors with his corps of assistants in the cause of hydro-therapy.

His institution is the mecca of thousands of afflicted from every country of the globe, including numerous Americans.

To see the brilliant results achieved there by hydro-therapy, diet, massage, rest and healthful exercise, must necessarily create in one the desire to try and achieve some little good with this truly wonderful therapeutic agent, to spread its truths and induce others to adopt it.

